# The civic engagement community participation thriving model: A multi-faceted thriving model to promote socially excluded young adult women

**DOI:** 10.3389/fpsyg.2022.955777

**Published:** 2022-09-15

**Authors:** Irit Birger Sagiv, Limor Goldner, Yifat Carmel

**Affiliations:** ^1^School of Creative Arts Therapies, Faculty of Welfare and Health Sciences, The Emili Sagol CATs Research Center, University of Haifa, Haifa, Israel; ^2^Department of Educational Counseling, Faculty of Education, Beit Berl College, Kfar Saba, Israel

**Keywords:** civic engagement, thriving, women, young adults, identity, community

## Abstract

Social policies to promote socially excluded young adult women generally concentrate on education, employment, and residence but tend to neglect thriving. The current article puts forward a Civic Engagement Community Participation Thriving Model (CECP-TM) that views thriving as a social policy goal in and of itself. It posits that civic engagement, beyond its contribution to social justice, serves as a vehicle for thriving through self-exploration and identity formation. Both are considered key components of successful maturation and thriving. Nonetheless, civic engagement and self-exploration tend not to be nurtured in socially excluded young adult women, a unique group experiencing intersecting discrimination. The model shows how active civic engagement in the context of a community of peers contributes to developing a sense of belonging and connectedness and promotes new self-reflection, identity formation, and agency capabilities. When situated within the context of intersectionality, these encourage the development of critical consciousness and new understandings of “who I am and how I fit into the social world in which we live.” These can provide a sense of meaning, contribute to identity formation, and promote the thriving of the self and the community. Several examples illustrate the model.

## Introduction

The social inequity associated with globalization underscores the need to confront social exclusion (Hazari and Mohan, 2015; Muñoz Arce and Pantazis, 2019). Social exclusion is defined as a state or a process in which individuals or groups are denied full participation in social, economic, and political life (Benbow et al., 2015; Hazari and Mohan, 2015) and has vast negative implications for individuals’ quality of life (Enderle, 2018). Despite making up more than half of the population, women experience greater social exclusion and inequality (Strier, 2010; Jensen and Arnett, 2012; Lightman and Good Gingrich, 2018). This gendered exclusion is expressed in women’s absence from the public sphere, limited involvement in civic/political life (Palència et al., 2017), and reduced participation in the labor market (Farre et al., 2020). These are reflected in lower income levels (Novo-Corti et al., 2014) and greater poverty (Fredman, 2016) compared to men. No less significant are women’s subjective experiences of exclusion, as manifested in decreased levels of belonging, self-esteem, and control (Choudhury and Kumar, 2021), as well as higher levels of stress, anxiety, and depression (Mata-Greve and Torres, 2020), which can endanger young adults’ wellbeing (Levy et al., 2020).

Extended young adulthood is one of the characteristics of today’s global, industrialized society (Webster et al., 2006; Wood et al., 2017) and now covers the period from 18 to 30 or even slightly beyond (Arnett et al., 2014; Scales et al., 2016). This stage is characterized by the postponement of adult obligations (i.e., getting a job, acquiring an education, and starting a family; Aronson, 2008; Harris et al., 2010), which enables prolonged self-exploration and a search for direction in life (Gutiérrez-García et al., 2018). However, since socially excluded young adults, mainly from lower socio-economic strata, face unique stressors in the transition to adulthood such as discrimination and higher poverty rates (Harris et al., 2010; Gutiérrez-García et al., 2018), they have fewer opportunities for self-exploration and undergo a much more rapid transition to adulthood (Harris et al., 2010; Bialik and Fry, 2019).

By shifting rapidly to participation in adult life and its associated obligations, they have fewer opportunities for self-exploration and self-definition (Webster et al., 2006; Webb et al., 2017). Hence, the process of identity formation is suspended or occurs in parallel to fulfilling adult roles and tasks (Oesterle et al., 2010; Hardy et al., 2013). This need to immediately embark on adult life is acerbated in terms of gender (Oesterle et al., 2010) since it imposes another dimension of exclusion on already complex life circumstances, which may deny women the opportunities to develop their capabilities, and the freedom to plan their lives (Greene and Patton, 2020). This situation adversely affects health, wellbeing, and thriving that extend into adulthood and can influence the next generation (Munford and Sanders, 2007; Hickey and du Toit, 2013).

Social workers, healthcare professionals, researchers, and policymakers worldwide are engaged in finding ways to promote young women’s successful passage to adulthood (Sonu et al., 2019). Nevertheless, in today’s Western, mostly neoliberal, economic context (Nikunen, 2016; Barnett and Bagshaw, 2020), public expenditures for social security services are scarce (Barnett and Bagshaw, 2020), and there is not enough social investment (Kuitto, 2016). Typical interventions targeting young adults (men and women) concentrate mainly on improving labor and working conditions (Webster et al., 2006; Nikunen, 2016), promoting housing solutions (Arthurson and Jacobs, 2004; Webster et al., 2006), enhancing education and training levels (Jordan, 2018), subsidies (Jordan, 2018), and supporting health and mental health conditions (Luchenski et al., 2018). Nevertheless, these welcome efforts have a minimal impact on inequality (Arthurson and Jacobs, 2004; Jordan, 2018). Researchers have suggested that concentrating on practical support and providing tangible basic needs (Chant, 2016) rather than directly addressing wellbeing as a social policy goal (Diener and Biswas-Diener, 2019) lessens the effectiveness of these interventions.

### Intersectionality as a key framework

To address women’s wellbeing differently, the current manuscript presents a multidimensional Civic Engagement Community Participation Thriving Model (CECP-TM) focusing on civic engagement and community participation to promote the thriving of socially excluded young adult women. The model draws on major critical post/feminist theories (Butler, 2013), which implement the notion of “intersectionality” as a key framework for designing interventions for socially excluded populations (Mojab and Carpenter, 2019). Intersectionality refers to the complex process and interactions between multiple identity dimensions of ethnicity, class, gender, and sexuality and their manifestations in experiences of marginalization, social disadvantage, and poorer health outcomes (Zambrana and Dill, 2009; Carbin and Edenheim, 2013; Cho et al., 2015). The concept of intersectionality aligns with theories of critical realism in rejecting the quest for a universal pattern of inequalities; instead, this framework seeks to unpack inequalities in social processes and uncover the subjective meaning of living in an intersectional position (Aronson, 2008; Bauer, 2014).

Originally, Black feminist scholars advanced intersectionality theory to account for multiple forms of subordination in the legal and political domains. Nevertheless, recently, intersectionality has gained increasing prominence in health studies that have examined how the health and wellbeing of various groups are impacted by structural oppression or marginalization (Cho et al., 2015). According to the intersectionality approach, multiple forms of oppression experienced by a single person have a cumulative effect that directly influences the individual’s internal sense of self and wellbeing. Therefore, to enhance wellbeing, the interlocking nature of multiple forms of oppression must be disentangled, so that the ways power dynamics at the macro-level of social systems and institutions interact with interpersonal relationships and subjective experience can be identified (Krumer-Nevo and Komem, 2015).

### Thriving as a core concept

Although intersectionality theory emphasizes the voices of people experiencing interlocking disadvantages, it does not restrict its focus to the experiences of suffering and oppression. Rather, it calls for identifying resilience and resources available in an intersectional social location (Earnshaw et al., 2013). Nevertheless, since a main developmental goals for disadvantaged young adult women is broadening future possibilities, returning to baseline while avoiding adverse outcomes (Ryff and Singer, 2003) does not capture their full potential.

Dominant theories in social welfare, humanities, and positive psychology have all advanced the concept of thriving to characterize better individuals’ wellbeing (Geldhof et al., 2013; Su et al., 2014). Thriving is defined as flourishing (growing or developing vigorously), prospering (accruing wealth or possessions), and progressing toward or realizing a goal despite the circumstances (Su et al., 2014; Diener and Biswas-Diener, 2019). Thriving views human development as a process and describes the aspiration for a very high living level and fulfillment of human potential (Sheldon, 2018).

Different researchers have divided the concept of thriving into various domains (Brown et al., 2017; Ross et al., 2020). The three prime dimensions are feelings of happiness, enjoyment, comfort, contentment, and the absence of distress (i.e., hedonic wellbeing), a sense of purpose and meaning in life, self-actualization, progress towards meaningful life goals, self-efficacy, agency, and control (i.e., eudaimonic wellbeing; Huta and Waterman, 2014; Sheldon, 2018), and engaging in deep, close, healthy relationships (i.e., social wellbeing; Feeney and Collins, 2015).

## The model

**Figure 1:**
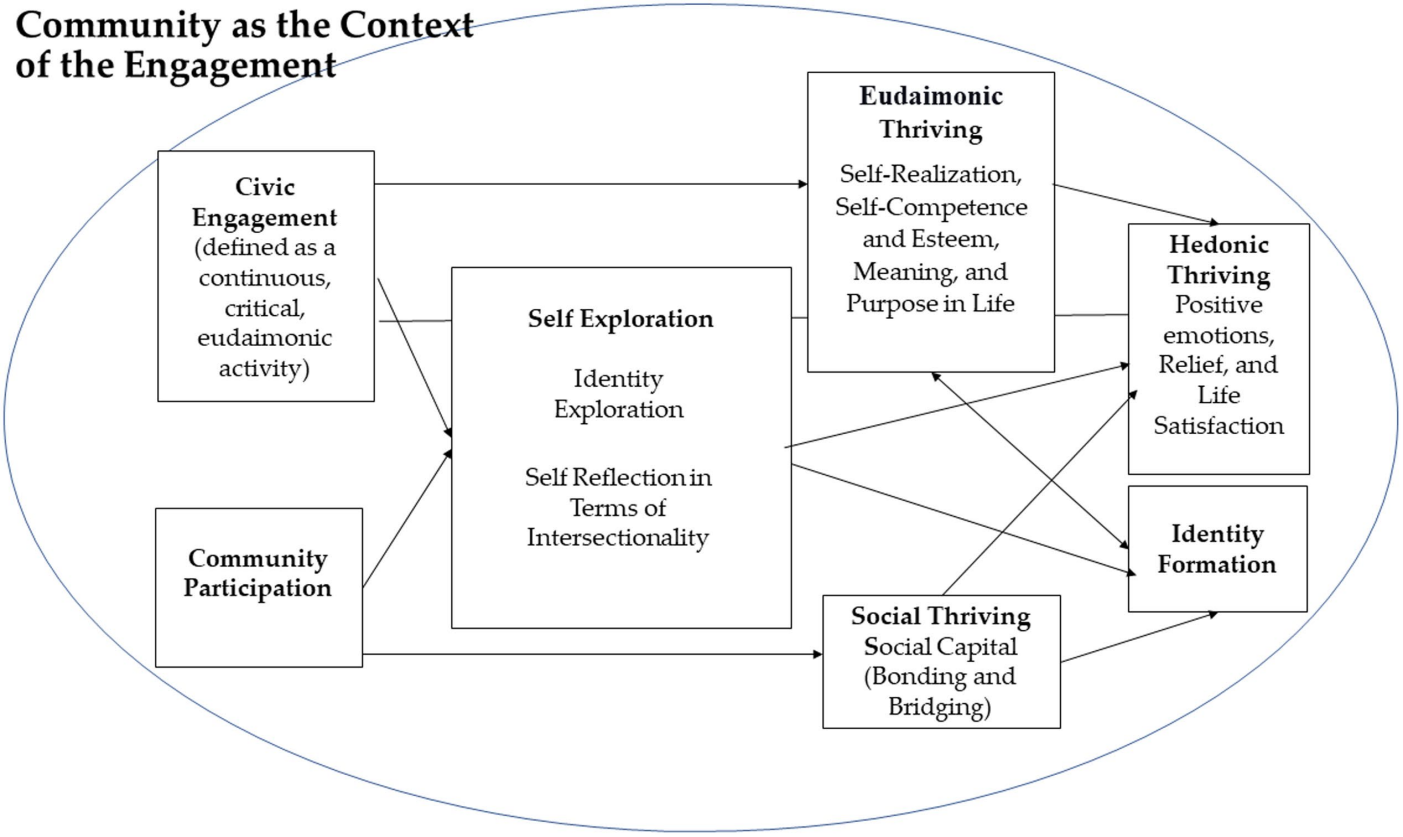
The civic engagement community participation thriving model (CECP-TM).

Built on the key concepts of intersectionality and thriving, the current article describes a multi-faceted developmental trajectory model, designed to promote the thriving of socially excluded young adult women, by participating in critical civic engagement program in a setting of a community of peers. The model is based on the premise that civic engagement in a community setting can directly augment these women’s thriving by promoting a sense of meaning and purpose in life (eudaimonic thriving). In addition, participating in a community was hypothesized to directly encourage marginalized women’s social thriving by providing them with the opportunity to establish deep and meaningful relationships and social networks (social thriving), as well as fostering their hedonic thriving by instilling positive emotions and life satisfaction. This model thus innovates by charting the indirect development towards thriving through the mediation of self-exploration (identity exploration and self-reflection in terms of intersectionality), which can lead to the fulfillment of all three thriving domains and promote these women’s identity formation. Specifically, social thriving contributes to identity formation, whereas eudaimonic thriving and identity formation mutually reinforce one another (see Figure 1).

Although the assumption that civic engagement promotes wellbeing and identity formation has often been explored (see, for example, Goldner and Golan, 2019; Markovich et al., 2019), few studies have examined the mechanisms through which civic engagement enhances wellbeing. Only a small number have provided a complete framework to describe the ways civic engagement promotes wellbeing and advances the eudemonic, hedonic, and social components of thriving when characterizing the contribution of civic engagement. In most cases, the literature has discussed the contribution of service-learning programs to undergraduate students (a relatively privileged sample). Even when less-represented female students have been the focus (see, for example, Goldner and Golan, 2019), these studies have been conducted in the context of campus-community partnerships. For instance, Markovich et al. (2019) examined campus-community partnerships operating in Israel while exploring the potential of these partnerships to facilitate transformative change in conflict zones. Although their book takes a feminist perspective and deals with how institutional hegemonic academic dynamics shape students’ identity, the authors mainly concentrate on how campus-community partnerships expose these dynamics to promote social change and advance human rights in conflict areas. Thus, identity exploration as a primary liberating vehicle to promote thriving is not the central theme.

### Civic engagement as a driver of thriving

Civic engagement refers to how individual and collective actions aimed at addressing issues of public concern are undertaken to improve conditions for others and/or help shape a community’s future. Engagement of this type can lead to a sense of connection, interrelatedness, and commitment to the community at large (Adler and Goggin, 2005; Vindhya, 2012; Fenn et al., 2021). Civic engagement includes any step intended to enhance the quality of life (Cnaan and Park, 2019). It can take many forms, from individual volunteering to organizational involvement, and from addressing a specific social issue and promoting social change to voting (Flanagan and Levine, 2010; Ballard et al., 2019). These acts can be regular or episodic and constitute one of the fundamentals of a democratic society that emphasizes active citizenship (Adler and Goggin, 2005; Ejlskov et al., 2014). Data indicate that women’s civic engagement is often situated in the private sphere, within their homes and neighborhoods, where they can express their voice in a secure atmosphere, before turning to larger civilian spheres (Godquin and Quisumbing, 2008; Jensen and Arnett, 2012; Jupp, 2017).

Overall, research findings are consistent with the notion that civic engagement can be highly beneficial, having a positive cumulative effect on individuals’ physical and mental health, life satisfaction, and thriving (Son and Wilson, 2015; Yeung et al., 2017). For example, a cross-sectional empirical study on students reported a strong relationship between civic engagement and wellbeing, mediated by service self-efficacy and meaning in life (Fenn et al., 2021). A review and meta-analysis demonstrated that volunteering positively affected depression, life satisfaction, and wellbeing (Jenkinson et al., 2013). Further empirical support for these findings emerges from a cross-sectional survey showing that in comparison to nonvolunteering, formal volunteering once a week was associated with twice the likelihood of thriving (Santini et al., 2019). Similarly, studies have repeatedly shown that civic engagement is a significant driver of young adults’ thriving (Jenkinson et al., 2013; Webb et al., 2017; Santini et al., 2019), generating self-efficacy and agency, and meaning in life.

Nonetheless, socially excluded young adults engage less than young people from higher socio-economic classes (Fenn et al., 2021), partly because such activities are too burdening for those who are already burdened (Fenn, 2022). However, Fenn (2022) comprehensive literature review suggests that an informed, tailored-to-need civic engagement activity can facilitate participation and bolster thriving (Fenn, 2022). Studies have shown that civic engagement can compensate for a lack of resources and development opportunities through exploration and helping others, which enables disadvantaged young adults to experience personal change, specifically in terms of resilience, identity, and social capital (Webb et al., 2017).

### Civic engagement as a generator of meaning in life, satisfaction in life and positive emotions

Studies on highly diverse samples implementing various forms of data collection suggest that significant sources of meaning in life can be derived from personal relationships, achievement, success, and altruism (Webb et al., 2017; Xi et al., 2022). Specifically, engagement at the macro-level (connecting with other members of one’s community) while addressing values, needs, goals and caring for others enables individuals to be involved in a eudaimonic activity that targets issues beyond the ego and mundane concerns. Hence, connecting to something more significant than the self (i.e., the eudaimonic component in wellbeing) is critical to advancing individuals’ wellbeing (Sheldon and Lyubomirsky, 2006; Nelson et al., 2016; Sheldon, 2018). By grappling with challenges and unexpected situations in new surroundings, individuals are called upon to show initiative and creativity and acquire new communication and leadership skills. These promote their sense of self-competence and lead to the realization of personal potential (Francis East and Roll, 2015; Fenn et al., 2021). This suggests that civic engagement facilitates a sense of meaning and purpose in life, which provides individuals with a crucial way to allocate their resources toward meaningful achievements that give a sense of satisfaction and happiness (i.e., hedonic wellbeing).

The idea that engaging in eudaimonic actions (highly meaningful, intentional, and beneficial behaviors) such as civic engagement can facilitate positive emotions is also based on Aristotelian philosophy as manifested in Sheldon (2018) Eudaimonic Activity Model. We suggest that for women who are not regularly exposed to such experiences in particular, civic engagement can be highly beneficial, by fueling them with positive emotions and a sense of enjoyment. This can have a cumulative effect which relieves their draining, alienating, and muted reality (Fenn et al., 2021) and can promote thriving both directly and indirectly (Ballard et al., 2019; Christophe et al., 2021).

### Community participation as a generator of social and hedonic thriving

Community participation is a broad and complex term describing interactions and collaborations among individuals with similar identities, goals, and interests. Through an ongoing process of participation, the identity of the participants and the community itself is continuously created (Strier, 2010; Christens and Speer, 2011), supporting the development of “social capital” (Brooks and Nafukho, 2006). Social capital is a term with many definitions relating to the amount and strengths of social connections stemming from family support, peer networks, and community stability (Pettit et al., 2011; Webb et al., 2017). It is customary to divide social capital into bridging and bonding, representing different levels of connectedness and the quality and/or quantity of social relations, both of which are increased through community participation (Christens and Speer, 2011; Landstedt et al., 2016).

Bridging social capital refers to establishing relationships between different groups of people, within and outside the community, characterized by weaker ties and often referred to as operational or general community participation (such as voting). These relationships can enable socially excluded young adults to extend their social networks, engage with meaningful organizations in their environments, and widen their mobilization opportunities, which constitute an important way to promote their future options and status (Moghadam, 2003). Bonding social capital refers to strong relationships between individuals within a homogeneous group (Pettit et al., 2011; Landstedt et al., 2016).

The development of close and meaningful relationships (i.e., bonding) promoted by participating in a community aligns with women’s inclination to group in what is classically termed “sisterhood” communities to promote women’s rights and involvement in the socio-political arena (Tesoriero, 2006; Francis East and Roll, 2015; Jupp, 2017; Andersen and Banerjee, 2019). These communities, characterized by trust, support, coalitions, collaborations, and effective communication (Taylor et al., 2000; Gittell et al., 2012; Webb et al., 2017), allow women to participate in decision-making about their future and improve their lives. In addition, these communities play a protective psychological role by reducing stress (Cicognani et al., 2009), lessening depression (Landstedt et al., 2016), improving social wellbeing (Cicognani et al., 2015), and at times people’s lifetimes (Holt-Lunstad and Smith, 2012).

Increasing community participation is an essential component of integration in a democratic society (Etzioni, 2011) which helps fight poverty and social exclusion. Thus, for socially excluded young adults, participating in a community that combines positive and meaningful relationships holds numerous benefits. These include strengthening their significance to others while augmenting a sense of worth, belonging, and connectedness. It also compensates for the lack of social resources crucial to young adulthood (Webb et al., 2017), serves to establish networks, and can help overcome experiences of adversity (Hartling, 2008; Soska et al., 2010; Arslan, 2019; Azpiazu Izaguirre et al., 2021). It thus enables the development of resiliency, positive emotions, and better health (Feeney and Collins, 2015; Dunkel Schetter, 2017). This is especially true for women, who use social networks (bonding and bridging) as a powerful resource for promoting social resistance and social rights (Francis East and Roll, 2015). In our model, participating in the community serves as the platform for civic engagement and a vehicle to catalyze women’s social and hedonic thriving.

### Identity exploration and intersectionality self-reflection as a generator for thriving and identity formation

Civic engagement and participation in the community can also shape the process of self-exploration and identity formation, the cornerstones of our model. Identity formation is often divided into exploration and commitment (Klimstra et al., 2010; McLean et al., 2016). Exploration is defined as actively engaging in targeted activities and searching for alternatives while trying to answer the question of ‘who am I?’. Commitment refers to making decisions about these alternatives while defining ‘myself’ (McLean et al., 2016). Identity formation is a core developmental process that begins in adolescence and continues into young adulthood, in which different dimensions of identity evolve towards greater maturity, forming a more stable identity in different content domains (ideological, interpersonal, etc.). It is characterized by individuals’ increased reflection on their certainty about their commitments (Klimstra et al., 2010; McLean et al., 2016).

Findings have indicated that individuals who have made identity commitments (i.e., decided that certain things are important to who they are), ideally through a process of exploration, have fewer mental health problems (e.g., anxiety and depression and suicidal self-injury; Crocetti et al., 2009), engage in fewer health-risk behaviors (e.g., alcohol use and sexual risk-taking; Bishop et al., 2005; Schwartz et al., 2013), have fewer mental health concerns and report higher levels of psychological wellbeing (e.g., self-esteem; Basak and Ghosh, 2008; Dunkel et al., 2011) and meaning in life (Hardy et al., 2013).

Our model suggests that civic engagement as a eudaimonic act, within a community setting can catalyze identity formation through a reflection on the intersection of the components of identity and identity exploration simultaneously. Specifically, it suggests that civic engagement can be experienced as a self-formative and maturational experience that can shape women’s identity and self-knowledge and serves as a springboard for shaping life goals and plans. Through civic engagement, women are called upon to explore new abilities and skills and fields of opportunities, explore career choices, examine new roles, and build a network of connections. In this respect, civic engagement may serve as a vehicle for acquiring social thriving reflected in social capital, which is especially important for individuals from socially excluded minority groups with fewer opportunities to be actively involved in identity exploration (Putnam et al., 2003; Strier, 2010).

Furthermore, interacting with people who differ in terms of age, social strata, and ethnicity from people that individuals encounter regularly, generates questions regarding sense of self, and may encourage women to explore their strengths and weaknesses. Hence, through their civic engagement, women can actively search for a more vigorous, sophisticated, and defined sense of subjectivity as to who they are and how they fit into their social world. Establishing a more robust subjectivity may also be enhanced through interaction with other women in the community, which can serve as a hall of mirrors, reflecting women’s abilities, assets, and difficulties (Goldner and Golan, 2019).

As a part of the change process, reflection on one’s civic engagement forms the common thread of the model because it emphasizes the importance of cognizing one’s eudaimonic activities, both personally (Christophe et al., 2021) and collectively (Wegner et al., 2019). Once women reflect on their upbringing, intersectional identities, assumptions, values, and restrictive conditions in which their civic engagement is enacted, critical social consciousness begins to form. This awareness helps them further analyze the world in which they live, and promote their self-determination, despite the systemic oppression they become more aware of (Ginwright and Cammarota, 2007; Goldner and Golan, 2019; Ajaps and Obiagu, 2020).

By applying an intersectional lens, disadvantaged women often recast their trauma as a motivation for taking on an active role and choosing the field of civic engagement (Jupp, 2017). Critical feminist thinking and intersectional theory both emphasize the need for socially excluded women to uncover how the components of their identity are differentially influenced and affected by social status, class, and ethnicity within a specific historical context (Bell et al., 2019; Biana, 2020). In this vein, research indicates that when civic engagement is viewed as a revolutionary act of the self in response to sociopolitical inequality, it can integrate into one’s identity in the form of resistance, thus forming an adaptive coping strategy that can promote eudaimonic thriving (Ginwright and Cammarota, 2007; Hope and Spencer, 2017).

More generally, researchers have suggested that interventions which involve civic engagement as a strategy to promote thriving are beneficial, especially when they implement social justice-based or critical civic engagement point of view (Hope and Spencer, 2017; Christophe et al., 2021; Fenn et al., 2021). Thus, women’s self-exploration that concentrates on the components of context, ideology, social structures, and power relations and extends beyond the practical aspects of acquiring skills and knowledge is likely to enable the construction of meaningful different identity components to serve as a broad platform for their self-realization and development (Daoud et al., 2012; Bairey Ben Ishay and Gigi, 2019; Goldner and Golan, 2019).

## Examples

In Israel, several projects and programs have been designed to enhance young adults’ eudaimonic, social, and hedonic thriving through civic engagement that promotes identity exploration in light of intersectionality. These include the Civic Service of Arab young adult women, which according to Yanay-Ventura et al. (2020) offers young Arab women the opportunity to crystalize their personal and citizenship identity. This takes place by exploring aspects of discrimination and marginalization that shape their identity and acquiring new skills and economic independence, thus bypassing the glass ceiling. The authors noted the concept of cultural capital and described how the participants unpacked various aspects of exclusion and discrimination internalized in their identity. These benefits are especially interesting given the ambivalent public attitude and opposition to participation in Arab communities (Yanay-Ventura et al., 2020).

Goldner and Golan (2019) describes the Israel Scholarship Education Foundation program (ISEF). This program operates civic-engagement social-educational projects, in which students from disadvantaged backgrounds regularly meet marginalized communities for two to 4 hours per week in return for a scholarship. The project aligns with the Foundation’s vision of reducing social gaps through higher education. The findings pointed to the significance of volunteers’ reflections on culture, ethnicity, and power distributions, which validated their cultural heritage and integrated intersectional aspects of their identity.

Below, we focus on the Young Women in the Lead - Social Activism in Young Adult Women’s Communities program, under the auspices of the Fund for Demonstration Projects of the Israel National Insurance Institute and the Gandyr Foundation. During this program, various organizations established 20 communities for socially excluded young adult women across Israel. These women’s communities are characterized by multiple marginalization, such as belonging to an ethnic minority group, residing in low-income peripheral towns, and lacking family support. In some cases, the women had a history of child abuse, neglect, or personal trauma, all amplified because of their gender.

In most cases, the women were recruited through welfare bureaus. The project aimed to encourage civic engagement as an innovative approach to promoting women’s wellbeing. The communities consisted of 5–10 young women who participated in weekly two-hour meetings in which designated coordinators introduced the women to issues such as civic engagement skills, social/political awareness, social justice, and gender. Participants were invited to be involved in building the community and initiate civic engagement that addresses social issues derived from their life stories and circumstances. Women were also invited to consciously reflect on matters of intersectional identity, social justice and gender throughout the program in group discussions guided by the communities’ coordinators.

The example below is taken from four in-depth semi-structured interviews with a 23-year-old Arab Israeli woman who participated in one of these communities for 3 years. Research assistants conducted the interviews in Arabic and later translated them into Hebrew. The interview consisted of questions about the community’s progress. These included: “What expectations did you have from participating in the project?” “If someone were to ask you to stop for a moment and look at your life to this day, how would you describe it?” “Can you describe how participating in the project has affected your life?” “Do you have plans for future jobs, education, leisure, family, and relationships?”

Asil (pseudonym) is a 23-year-old Arab Israeli woman living in a large Muslim-dominated city in the north of Israel. She was asked to recollect her motivations for joining a civic engagement community. Her response revealed a life full of hardship and despair, leading her to hope and wish for change.

I have gone through many periods of hardships in my life, and I am still going through them. Sometimes there are moments of despair when you are sure nothing will work out, and you are exhausted. But these don’t last. They are just bits of the time; afterward, you return to the struggle.

Nonetheless, this wish for change has little hope of coming to fruition, as shown by her sense of ambivalence and hesitation. “At first, I hesitated; my mother said, ‘you will not lose anything, try.’ So, I joined the community here.”

She nevertheless made it clear that something was profoundly missing by saying, “After graduation, I had a lot of free time. I felt like I was not really me. I wanted a change in life, in my personality, a break from the routine, to breathe a little, just to go out! To feel independent and see beyond routine.”

Later, Asil talked about the importance of being part of a supportive community of women, gaining confidence, and exploring her capabilities. She repeatedly used the terms “voice” and “voicing the self” as constituting significant steps in acquiring knowledge about the self and building her identity. She recalled: “I remember at first I was timid. I could not express myself.” Later on, she dared to express herself, especially in less conventional fields in her milieu.

I’m glad I joined, I found a place to express my voice, to speak freely about topics that are not usually discussed, it was very empowering that the group is for girls, and you can both give and receive, be a voice for those who do not have enough voice to speak and express themselves.

She continued by defining civic engagement as “…the possibility to give to each person, every little thing that can help, support, and promote,” thus linking the processes of civic engagement with personal and social change. In fact, she saw these three processes as almost identical:

It first and foremost starts from the inside. It does not matter for whom or for what. The main thing is that you stand up to support yourself, take care of yourself and thus others as well. I come here on my day off, without many hours of sleep, so yes, I am an activist… I am a little different each time I come here: sometimes I am calm, sometimes nervous, sometimes talking, sometimes quiet, etc. And I know that I am accepted as I am. And acceptance is mutual of course. That in itself is activism. Accepting the other allows for free, authentic, and the truest expression of the self.

In addition, she described her identity development associated with eudemonic thriving (i.e., findings meaning in the social change she is involved in) and the joy (i.e., hedonic thriving) associated with this development:

I personally have developed a lot… I feel growth… I am very happy… Creating change, talking about rights, about women, bringing things to light and not hiding them as is customary in my culture. It is exciting to understand that my participation is important; that I can contribute another voice, another opinion, another question… Another idea of being part of something

In terms of change and self-exploration, Asil described the persistent gap between her aspirations for identity exploration and her limited possibilities and choices. In her view, this gap began when she graduated from high school and started thinking about “what I would like to do and not just what I need to do,” even though “I do not see why there are things that prevent me from thinking and dealing with these thoughts and questions, but when I want to act sometimes I have to convince those around me and negotiate with my immediate environment (family).” These limitations relate to her social positioning, which she perceives through self-reflection in the light of intersectionality. Asil gained an understanding of this gap which is related to her life circumstances and society in general, and this has allowed her to broaden her perspective, make different interpretations, and engage in a range of actions, as she courageously described:

The community has been by my side through a process of self-awareness and connection to the self. Today I know Asil better (who I am, what I am, what I want …) more than ever. I am connected to who I am and believe in everything I am. I will give you an example: one day I had to go to the city, and on the way, I saw a man beating a woman, I was stressed, I cried, but nevertheless, I had the courage to call the police and report it. I felt really proud that I have the ability to defend, to express, to speak, not to be silent. I did not use to be like that. I did not have that courage. The community is a big part of all the inner changes I have gone through.

At the end of the final interview, Asil summarized her 3 years of participation. Her statements illustrate how the components of the model are combined and interconnected. She related to the importance of community in instilling feelings of connectedness and serving as a safe place for self-exploration in a secure atmosphere which allows her to contemplate and shape a different way of thinking. She repeatedly used the word “change” to describe her internal changes and the social changes in which the participants evolved. She described the community as a group of feminist women, changing their reality. She stated:

This project helped me a lot. I do good and receive good. I feel a sense of satisfaction that I am active, and I am in, I am for others. The group is like a warm home where I can express parts of myself that I cannot express anywhere else… My thoughts have taken on a slightly different shape, sometimes a slightly different turn… Giving, support and change. I am a girl who believes in change: changes in people, changes in the environment, personal change. The project was a place for unloading energies, thoughts, and deliberations. All the girls are willing to help and promote change in any way possible. The sense of belonging was strengthened several times over. Especially in the group and when it comes to women, girls and feminism … Planning a path in life, achieving goals by planning ahead. If I have a setback, I stop, I ask myself the relevant questions, get more information, turn to the questions, and start re-planning.

Another example is Hadil, a 20-year-old Arab Israeli woman living in a medium-sized village in the north of Israel. In response to a question about her interest in wanting to join the community to be involved in civic engagement, she talked about the importance of a supportive women’s community. She described wanting to create a secure space for her and others to promote change through civic engagement, referring to the internalization of discrimination and social control by the women themselves.

She said: I am in favor of women changing, working for peace… first and foremost among the women themselves. We are very oppressive of each other when we criticize and censor each other out of social conventions that we ourselves reinforce. Women are supposed to have each other’s backs. This is where activism begins, and this is where change really begins.

In a later interview, she talked about her growing feelings of connectedness and belonging in the community fostered by the women and the community’s coordinator. She described how these feelings catalyzed her motivation for active civic engagement, which led to meaning and purpose in life. Hadil also provided a sense of the indirect connection between civic engagement and community participation with identity exploration.

I feel and see that many things have changed since joining the community. Let’s start with the fact that there is support, the girls support me, and my aspirations and I support them. It is very encouraging. Beyond that, I am more active, I go out into the community to act, I started volunteering at school, I participate in community projects. I have power, I feel I have meaning, and I have a purpose… I’m important, and the fact that I can give makes me feel even more powerful. This is what encouraged me to want to get more education, expand my horizons and continue to be active for others and for myself. It makes a sense of vitality and hope.

Hadil elaborates how being a part of a group of women who share experiences, ideas, and actions regarding women’s inequality, led her to reflect on her own life in the light of intersectionality, and experience self-change. She described taking a more critical point of view on the condition of women in her community, being more empathic, caring more for others. She describes many good feelings, which are related to being a part of something and being active towards something novel, within her reach.

The activity enabled me to see a truer picture of women in society and the oppression of women. The group activity made me look at people more empathetically, while thinking of more options. Before I joined the community, I was not interested in what was happening in the village or the conflicts we were facing. Today, I feel more caring and interested in participating in the change. Today I feel it is my duty to understand, know, and take an active role in repairing and improving. Before joining the community, I saw myself as an ordinary girl living for ordinary needs and normal roles. Today, I see myself as more vital. I do not just exist to exist. I exist for action to be part of and for a goal.

## Summary and implications

Our theoretical model suggests a trajectory of subjective thriving through participating in community civic engagement *via* the mechanism of self-exploration and intersectionality. Although this kind of civic engagement differs from conventional civic engagement in young adults that evolves spontaneously, policymakers, third-sector organizations, institutions involved in service learning, and other welfare and health practitioners working with underprivileged populations can draw on this model to promote thriving among socially excluded women and other populations. This could involve developing white papers and plans for program interventions that decision-makers can use to allocate budgets and resources to establish civic engagement communities for socially excluded populations.

For civic engagement to be perceived as meaningful, attention should be paid to educating young women on how their personal lives can be translated into community goals and concerns. One way to do this is for social workers and field practitioners to guide and support the women in transforming their broad social goals into a series of small but meaningful acts of civic engagement to avoid despair and maintain motivation. Time and effort should be devoted to teaching women civic engagement skills and providing them with a solid background. Efforts should be made during community meetings to develop political awareness and critical thinking that can enable women to reflect on their identity in terms of the premises of social justice. Engaging in critical civic engagement that fights institutionally structured power dynamics which curtail women’s rights may encourage young women to express their silenced voices and re-consider their intersectional experiences. They can then actively address social concerns from the agent’s perspective rather than that of the victim while considering the issues of “equality” and “patriarchy,” which can restore their sense of agency and foster a liberating anti-oppressive standpoint of engagement.

Social workers and healthcare professionals should be aware that when prolonged civic engagement addresses personal and social needs and is analyzed in terms of gender and identity, it can become an internalized element in women’s identity that can promote resiliency and thriving. Social work curricula would benefit from incorporating thriving-informed interventions that go beyond subsidies, better housing, scaffolding education, and professional training, and should offer courses centered on paradigms targeting wellbeing and positive development trajectories. This transformation would help disadvantaged populations more fully develop their potential in their communities despite structural inequality and intersectional discrimination.

## Data availability statement

The original contributions presented in the study are included in the article/supplementary material; further inquiries can be directed to the corresponding author.

## Author contributions

IS and LG have developed and formulated the model, wrote the manuscript, and directly contributed to the work and its approval for publication. YC revised the draft of the manuscript.

## Funding

This work was supported by the Fund for Demonstration Projects of the Israel National Insurance Institute.

## Conflict of interest

The authors declare that the research was conducted in the absence of any commercial or financial relationships that could be construed as a potential conflict of interest.

## Publisher’s note

All claims expressed in this article are solely those of the authors and do not necessarily represent those of their affiliated organizations, or those of the publisher, the editors and the reviewers. Any product that may be evaluated in this article, or claim that may be made by its manufacturer, is not guaranteed or endorsed by the publisher.
